# Raster microdiffraction with synchrotron radiation of hydrated biopolymers with nanometre step-resolution: case study of starch granules

**DOI:** 10.1107/S0909049510028335

**Published:** 2010-09-03

**Authors:** C. Riekel, M. Burghammer, R. J. Davies, E. Di Cola, C. König, H.T. Lemke, J.-L. Putaux, S. Schöder

**Affiliations:** a European Synchrotron Radiation Facility, BP 220, F-38043 Grenoble Cedex, France; b Paul Scherrer Institut, Bioenergy and Catalysis Laboratory, CH-5232 Villigen PSI, Switzerland; c Centre for Molecular Movies, Niels Bohr Institute, University of Copenhagen, Universitetsparken 5, DK-2100 Copenhagen, Denmark; d Centre de Recherches sur les Macromolécules Végétales (CERMAV-CNRS), BP 53, F 38041 Grenoble Cedex 9, France

**Keywords:** biopolymers, nanometre raster-diffraction, radiation damage, starch granules

## Abstract

X-ray radiation damage propagation is explored for hydrated starch granules in order to reduce the step resolution in raster-microdiffraction experiments to the nanometre range. Radiation damage was induced by synchrotron radiation microbeams of 5, 1 and 0.3 µm size with ∼0.1 nm wavelength in B-type potato, *Canna edulis* and *Phajus grandifolius* starch granules. A total loss of crystallinity of granules immersed in water was found at a dose of ∼1.3 photons nm^−3^. The temperature dependence of radiation damage suggests that primary radiation damage prevails up to about 120 K while secondary radiation damage becomes effective at higher temperatures. Primary radiation damage remains confined to the beam track at 100 K. Propagation of radiation damage beyond the beam track at room temperature is assumed to be due to reactive species generated principally by water radiolysis induced by photoelectrons. By careful dose selection during data collection, raster scans with 500 nm step-resolution could be performed for granules immersed in water.

## Abstract 2

Radiation damage propagation was examined in starch granules by synchrotron radiation micro- and nano-diffraction techniques from cryo- to room temperatures. Careful dose limitation allowed raster-diffraction experiments with 500 nm step resolution to be performed.

## 1. Introduction

Raster-microdiffraction with synchrotron radiation micro­beams has become a routine technique for studying hierarchically organized synthetic and biological polymers (Riekel, 2000 ▶; Paris, 2008 ▶; Riekel *et al.*, 2009 ▶). Experiments are often performed at room temperature (RT) under *in situ* conditions. The ultimately accessible length scale in a raster-diffraction experiment is limited by the beam-size-defined step-resolution. A nanometre step-resolution allows in principle local inhomogeneities to be resolved in real space on length scales which are accessible in reciprocal space to high *Q*-resolution scattering techniques. In practice, a ∼200 nm step-resolution has been demonstrated for radiation-hard poly(*p*-phenylene terephthalamide) fibres (Müller *et al.*, 2000 ▶; Roth *et al.*, 2003 ▶). For hydrated biopolymers, such as cellulose or starch, step increments of 4–5 µm have been used at ∼13 keV in order to avoid spill-over of radiation damage to neighbouring raster-points (Schoeck *et al.*, 2007 ▶; Gebhardt *et al.*, 2007 ▶; Lemke *et al.*, 2004 ▶). This is somewhat larger than the travel range of photoelectrons in organic matter (O’Neill *et al.*, 2002 ▶) which is at the origin of secondary radiation damage (see below). We note that a similar strategy of separating irradiated and unirradiated regions has recently been proposed for protein crystal data collection (Stern *et al.*, 2009 ▶). In order to extend step-resolutions to the nanometre range by using now routinely available nanobeams (Riekel *et al.*, 2009 ▶), a better understanding of the propagation of radiation damage in hydrated biopolymers is required. The discussion of radiation damage in biopolymers can be based on the current understanding of radiation damage in protein crystals (Ravelli & Garman, 2006 ▶; Garman, 2010 ▶; Nave, 1995 ▶). Indeed, the absorption of a photon by an atom resulting in its ionization and the instantaneous ejection of an inner-shell photoelectron (photoelectric effect) is the dominating cause for ‘primary’ radiation damage at incident photon energies around 13 keV. The primary photoelectron has a beam track of a few micrometres at ∼13 keV (O’Neill *et al.*, 2002 ▶) and can induce up to 500 ‘secondary’ photoelectrons with an energy spectrum extending to thermalization. Model simulations suggest that for X-ray beams of ∼1 µm and smaller most of the energy of the primary photoelectron will be deposited outside the beam track (Moukhametzianov *et al.*, 2008 ▶). These photoelectrons induce ‘secondary radiation damage’ effects through ionization and excitation events. The creation of reactive species by radiolysis of water is an important contribution to secondary radiation damage in hydrated protein crystals (Henderson, 1990 ▶; Holton, 2009 ▶; Ravelli & Garman, 2006 ▶; Nave, 1995 ▶; Garman, 2010 ▶). Among the reactive species generated are hydrated electrons and radicals such as OH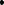 (Ward, 1988 ▶) which are known to attack polypeptide chains through H-atom abstraction from N atoms (Rao & Hayon, 1974 ▶) and polysaccharides with hydrogen abstraction at C^1^ and C^4^ (Ershov & Isakova, 1987 ▶). Cryocooling is used routinely in synchrotron radiation protein crystallography to immobilize the heavier reactive species, hence reducing structural loss by secondary radiation damage effects (Hope, 1988 ▶; Nave & Garman, 2005 ▶; Teng & Moffat, 2002 ▶). Hydrated electrons remain, however, mobile down to a few K (Dick *et al.*, 1998 ▶).

We explore in this article primary and secondary radiation damage effects in several B-type starch granules by synchrotron radiation microdiffraction techniques. The strong hydration capability of the polysaccharide chains in starch makes it a good model system for studying the formation of radiolytic products in hydrated biopolymers. Indeed, B-type potato starch is capable of absorbing about 30% water from saturated water vapour (Buléon *et al.*, 1982 ▶) while protein crystals contain roughly between 20 and 80% water. In addition, several raster-microdiffraction studies on single B-type starch granules have already addressed the local structure of the polysaccharide chains (Buléon *et al.*, 1997 ▶; Waigh *et al.*, 1997 ▶; Lemke *et al.*, 2004 ▶; Chanzy *et al.*, 2006 ▶; Gebhardt *et al.*, 2007 ▶) and their superstructure (Waigh *et al.*, 1999 ▶).

## 2. Experimental

### 2.1. Starch granules

**Figure 1:**
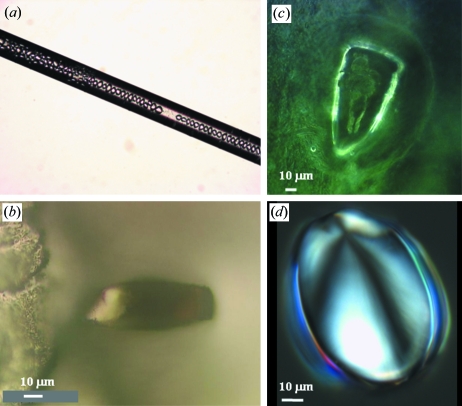
(*a*) Potato starch granules in a 100 µm-diameter borosilicate capillary filled with water. (*b*) Optical micrograph of a 12 µm-thick laser-cut section from the centre of a dry potato starch granule attached to a glass support by beeswax; (*c*) *Phajus grandifolius* granule in a glass capillary saturated with water vapour. A water drop has condensed on the granule. (*d*) Polarized-light optical micrograph of a *Canna edulis* granule showing the location of the growth centre.

We used B-type starch granules from potato (Lemke *et al.*, 2004 ▶), *Phajus grandifolius* (Chanzy *et al.*, 2006 ▶) and *Canna edulis* (Hall & Sayre, 1970 ▶) which are readily available in dimensions of 50–100 µm (Figs. 1*a*–1*d* ▶). This allows experiments to be performed at different temperatures and radiation doses on the same granule which simplifies the reduction and interpretation of data. Note that the diffraction patterns of hydrated potato starch (Lemke *et al.*, 2004 ▶) and the other two B-type starch species used in this work are identical (see supplementary information^1^).

Experiments were performed on whole granules and sections from the central part of potato starch granules. Granule sections allow the propagation of radiation damage to be studied without the influence of the granule shell structure (Buléon *et al.*, 1998 ▶). The approximately 25 µm-thick sections were prepared by laser micro-dissection (Seidel *et al.*, 2008 ▶; Davies *et al.*, 2008 ▶) [Fig. 1(*b*) ▶; see also supplementary information]. Granules were kept at RT in sealed borosilicate or quartz capillaries saturated with water vapour or filled with water (Fig. 1*a*, 1*c* ▶). Capillaries containing humidified cellulose tissue showed condensation of water drops around the granules so that total immersion can be assumed (Fig. 1*c* ▶). For experiments at 90 or 100 K, granules or granule sections were soaked in a ∼30% ethylene glycol/water solution and transferred into nylon cryoloops (Fig. 1*b* ▶). Flash-freezing of granules by a nitrogen cryoflow system has been described elsewhere (Lemke *et al.*, 2004 ▶). The cryoflow system was also used for maintaining specific temperatures up to 273 K (see also supplementary information).

### 2.2. Synchrotron radiation experiments

The different beam conditions and experimental set-ups used for data collection reflect advances in beamline instrumentation over successive experiments. Experiments were performed with a monochromatic beam at a wavelength of ∼0.1 nm. The absolute photon flux was determined by a calibrated photodiode at the sample position. A focal spot of 5 µm, corresponding to a flux of about 10^11^ photons s^−1^, was obtained by the combination of parabolic Be refractive lenses and collimator (Chanzy *et al.*, 2006 ▶). Focal spots of 1 µm and 0.3 µm, corresponding to a photon flux of ≤4 × 10^10^ photons s^−1^, were produced by Kirkpatrick–Baez mirror focusing (Riekel *et al.*, 2009 ▶). All quoted flux values correspond to 200 mA storage ring current.

For data collection, three different detectors were used. The two CCD-based detectors were a MAR165 and a FReLoN CCD camera (Labiche *et al.*, 2007 ▶). Both operate with 2048 × 2048 pixels and 16-bit readout. For fast raster-diffraction experiments, without readout noise, a Medipix2 pixel detector was employed with a single detector chip of 256 × 256 pixels (Ponchut *et al.*, 2002 ▶; Graceffa *et al.*, 2009 ▶). Further instrumental details are provided in the supplementary information.

### 2.3. Structural loss

Starch is a semicrystalline carbohydrate biopolymer composed of a shell structure containing amorphous and semicrystalline growth rings (Buléon *et al.*, 1998 ▶). The absolute crystallinity of hydrated starch granules is not well known, with values of 25–40% derived from X-ray diffraction experiments on potato starch (Buléon *et al.*, 1998 ▶). The formation of a crystalline B-type starch fraction during hydration is linearly correlated with the increase of the intensity of the strong 100 reflection (*d* ≃ 1.54 nm), reflecting the lateral chain–chain correlation of the hydrated fraction (Buléon *et al.*, 1982 ▶; Lemke *et al.*, 2004 ▶). The reduction of 100-reflection intensity upon irradiation is used for quantifying the relative change of crystallinity. Full crystallinity (*i.e.* 100% relative crystallinity) is assumed for starch granules in saturated water vapour or immersed in water. Complete amorphization (*i.e.* 0% crystallinity) corresponds therefore to the total disappearance of the 100-reflection.

In this study, radiation damage will be expressed in photons nm^−3^. We note the existence of more refined criteria for structural loss in protein single crystals taking the completeness of resolution shells into account (Teng & Moffat, 2000 ▶).

We used a combination of the *FIT2D* software application (Hammersley, 2009 ▶) and specialist batch processing software (Davies, 2006 ▶) for data analysis. Extended raster-scans of granules are displayed as composite images with ‘pixels’ composed of diffraction patterns or scaled to the intensity of a particular reflection.

## 3. Results and discussion

### 3.1. *In situ* study of radiation damage

**Figure 2:**
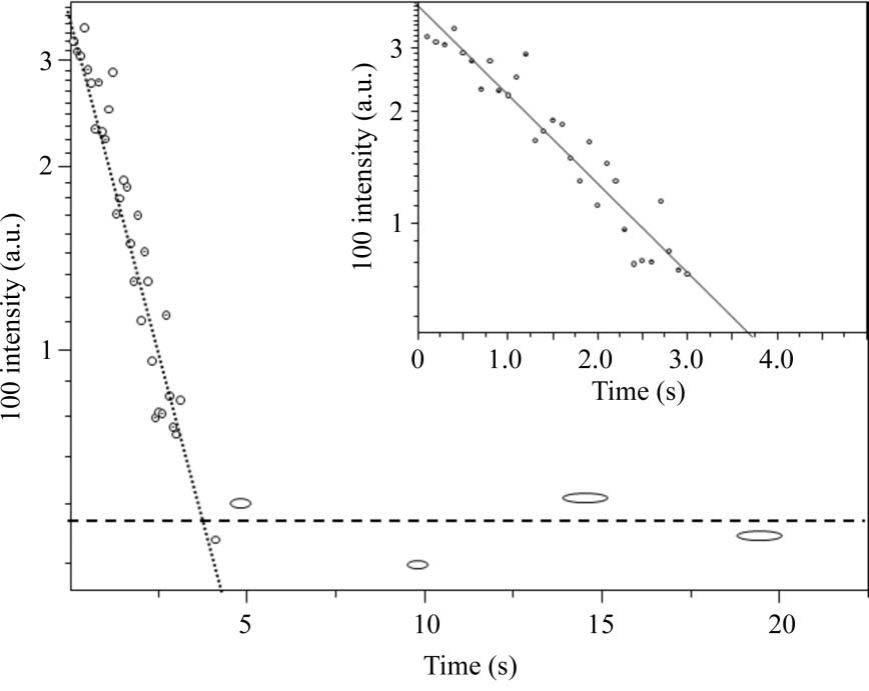
Decay of the 100-reflection at RT for a potato starch granule immersed in water and irradiated with a 1.1 µm beam. Several data points have been averaged for *t* ≥ 5 s.

We quantified radiation damage for a single 50 µm-diameter potato starch granule contained within a water-filled capillary at RT (Fig. 1*a* ▶) which was exposed at the same position to 200 exposures of 0.1 s each by a 1.1 µm beam. The total data acquisition time (including readout time of the FReLoN detector) was 158 s. The intensity decay rate of the 100-reflection (to <20% residual intensity) follows a first-order rate law without a lag in the onset of radiation damage (Fig. 2 ▶). The photon beam flux of 1.8 × 10^10^ photons s^−1^ corresponds to a dose of 1.3 ± 0.1 photons nm^−3^ for a complete (extrapolated) structural loss. This is a factor of about four times smaller than the value of ∼5 photons nm^−3^ determined previously (see supplementary information) (Buléon *et al.*, 1997 ▶). This could be due to an increased radical concentration in the crystalline fraction owing to an increased water content as compared with granules used in the previous study which were kept only in saturated water vapour (Buléon *et al.*, 1997 ▶). We also note T_2_ NMR results suggesting an increase of free mobile water with respect to bound water for the highest water content (Lechert *et al.*, 1980 ▶). The origin of the residual 100-reflection intensity in Fig. 2 ▶ is not resolved until now. It is possible that the slower degradation kinetics at longer times is linked to the internal architecture of a starch granule which is assumed to consist of a few hundred nanometre-sized blocklets (Gallant *et al.*, 1997 ▶). A fraction of larger blocklets could degrade slower, as reported for enzymatic attack (Gallant *et al.*, 1992 ▶, 1997 ▶). Particle size analysis during amorphization (determined according to the Scherrer formula from the 100-reflection) shows too much fluctuation to test this hypothesis. This is compounded by reports on a more complex shape of the 100-peak owing to the presence of two hydrated fractions (Lemke *et al.*, 2004 ▶) which makes a particle size determination problematic.

### 3.2. Temperature dependence of radiation damage

**Figure 3:**
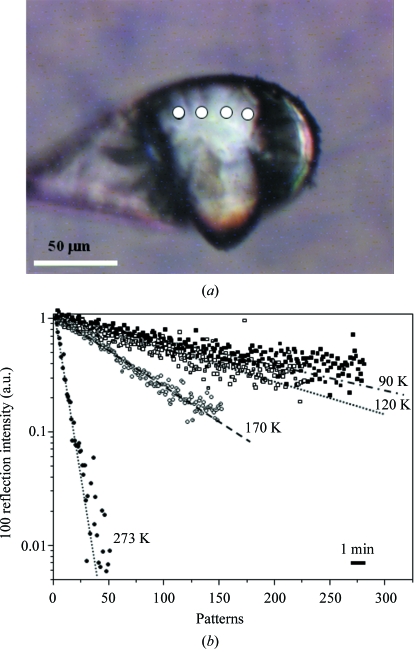
(*a*) *Phajus grandifolius* granule in a nylon cryoloop under N_2_ cryoflow conditions. Circles mark the position of the beam on the granule where the kinetics of loss of 100-intensity was measured as a function of temperature. (*b*) Kinetic curves for *Phajus grandifolius* measured with 0.5 s data collection per point and (on average) 3.7 s readout time per point. The data points for each temperature were scaled to *I*_100_ = 1.0 at *t* = 0 and fitted by a linear regression function. For a typical diffraction pattern with the 100-reflection, see Lemke *et al.* (2004 ▶).

In order to verify the presence of primary and secondary radiation damage, the temperature dependence of structural loss for a *Phajus grandifolius* starch granule was studied. The reduction in the 100-reflection intensity at a specific temperature was monitored during sequential exposures at a selected position on a single granule (marked by circles in Fig. 3*a* ▶). This procedure was repeated at four different temperatures, between 90 K and 273 K. The resulting decay in reflection intensity reveals that the rate of structural loss depends approximately linearly upon temperature (Fig. 3*b* ▶). Whilst the decay rate for the 90 K and 120 K data are almost identical, an increased decay rate is observed at 170 K and 273 K. A similar behaviour has been reported for protein crystals where radiation damage appears to be insensitive to temperature below about 150 K. This effect has been attributed to primary radiation damage, as the motion of radiolytic products contributing principally to secondary radiation damage effects is mostly frozen-in (Teng & Moffat, 2002 ▶). The acceleration of radiation damage in the *Phajus grandifolius* granule at 170 K (Fig. 3*b* ▶) can therefore be related to secondary radiation damage owing to the onset of diffusion of heavier radicals as in protein crystals (Teng & Moffat, 2002 ▶).

### 3.3. Radiation damage in granules irradiated at 100 K

**Figure 4:**
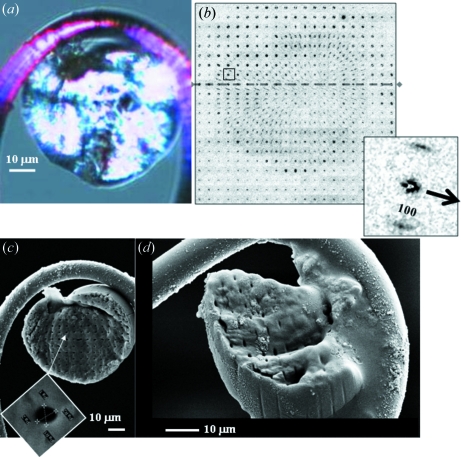
(*a*) Polarized-light optical micrograph of a hydrated potato starch granule section showing a Maltese cross. (*b*) Composite image composed of ‘pixels’ consisting of diffraction patterns limited to the 100-reflection. A single pattern with the direction of the local fibre axis indicated by an arrow is shown. The patterns were recorded during a raster scan with 4 µm steps of the granule section shown in the same orientation as in Fig. 5(*a*) ▶. The patterns of the line-scan through the growth centre are discussed in the supplementary information. (*c*) SEM image of the same granule section after the synchrotron radiation raster experiment. The inset shows the zoomed hole of a single track with dimensions 0.87 µm (PA1-PAR1), 0.97 µm (PA2-PAR2). The orientation of the zoomed hole is the same as in the full image. (*d*) SEM image of a second raster-scanned granule section. The partially broken section has been tilted to make the tracks running through the whole section visible at the edge.

Protein microdiffraction on a Xylanase II crystal with a ∼1 µm beam at 100 K suggests a restriction of radiation damage to the beam track which can be understood by an escape of the majority of photoelectrons from the irradiated volume (Nave & Hill, 2005 ▶; Moukhametzianov *et al.*, 2008 ▶). We verified this effect for a ∼25 µm-thick section from a hydrated potato starch. The optical microscope image obtained with crossed polarizers of the granule section shows birefringence owing to the radial orientation of chains, lying in the plane of the cut surface (Buléon *et al.*, 1998 ▶; French, 1972 ▶) (Fig. 4*a* ▶). The granule section was raster-scanned with a ∼1 µm synchrotron radiation beam oriented normal to the cut surface with 4 µm step-increments at 100 K. The exposure time of 10 s per raster point corresponds to an accumulated dose of approximately 8 photons nm^−3^ which is sufficient to destroy the local structural order at RT but not at 100 K (Lemke *et al.*, 2004 ▶).

The azimuthal width of the 100 reflection observed in a transmission microdiffraction experiment is sensitive to the granule shell structure as the polysaccharide chains are oriented normal to the shell surface (Lemke *et al.*, 2004 ▶). Indeed, a fibre texture observed at the granule edge is gradually transforming into a powder texture towards the centre of the granule (Lemke *et al.*, 2004 ▶). In contrast, a fibre texture is observed for most of the granule section in agreement with the polarized microscopy results (Fig. 4*b* ▶). The local fibre axes point towards several patterns with a broad azimuthal 100-distribution, which correspond also to the origin of the Maltese cross in Fig. 4(*a*) ▶ and are therefore assigned to the disordered growth centre (also called *hilum*).

A scanning electron microscopy (SEM) image of the irradiated section reveals holes at the position of the individual raster-points (Fig. 4*c* ▶). The diameter of an individual hole is determined from the SEM image as 0.97 µm × 0.87 µm (horizontal × vertical) (inset image in Fig. 4*c* ▶). This value is in good agreement with the beam size derived by knife-edge scans. The hole separation also matches the 4 µm × 4 µm mesh, at least within the positioning accuracy of the translation stages. The micrograph of a tilted section from a second raster-scanned granule shows that the beam tracks pass completely through the granule, as already observed previously for a larger beam (Chanzy *et al.*, 2006 ▶) (Fig. 4*d*). This result suggests that the primary radiation damage remains confined to the beam track at cryotemperatures. We tentatively assume that primary radiation damage owing to atomic ionization results in a cleavage of the gluco-pyranose ring followed by a further decomposition into fragments including gaseous products. Such decomposition reactions are known from γ-irradiation of cellulose and starch (Ershov & Isakova, 1987 ▶) and can explain the hollow tracks observed by SEM (Figs. 4*c* and 4*d* ▶). The SEM image does not, however, reveal visual evidence for secondary radiation damage effects induced by secondary photoelectrons propagating beyond the X-ray beam track (Moukhametzianov *et al.*, 2008 ▶) and generating reactive species such as OH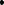, H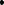 and hydrated electrons through radiolysis processes (Ravelli & Garman, 2006 ▶; Garman, 2010 ▶). The heavier radicals are, however, immobilized at 100 K and do not contribute to secondary radiation damage. Only hydrated electrons remain mobile at cryotemperatures (Dick *et al.*, 1998 ▶) and can result in hydrogen-abstraction at C^1^ and C^4^ with subsequent chain-scission effects (Ershov & Isakova, 1987 ▶). We note that radiation-induced changes of unit-cell and crystal symmetry in A-amylose crystals at cryotemperatures have also been attributed to chain-scission effects (Popov *et al.*, 2006 ▶). As both recrystallized amylose and the crystalline fraction of B-type native starch are composed of double-helical polysaccharide chains (Buléon *et al.*, 1998 ▶; Imberty & Pérez, 1988 ▶), similar radiation effects can be assumed to exist in both materials.

### 3.4. Radiation damage in granules irradiated at RT

We explored radiation damage effects by mobile reactive species for granules which were kept in a quartz capillary saturated with water vapour. A specific position on a single granule was exposed to an X-ray microbeam at RT for a fixed time and then raster-diffraction with short exposures was carried out around the irradiated area. The exposure time of a single diffraction pattern corresponded to a fraction of the dose required for complete structural loss which allows radiation damage spill-over onto neighbouring raster-points to be avoided. The raster-scan range was limited so that specific doses could be applied at several locations on the same granule.

**Figure 5:**
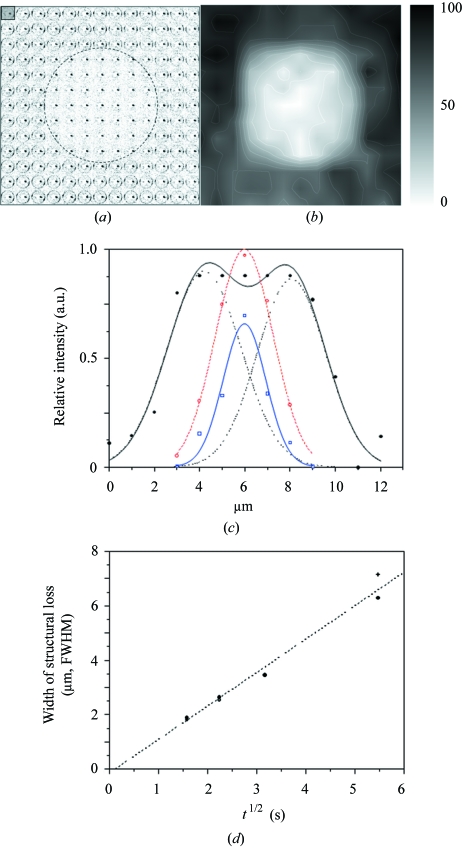
(*a*) 13 × 13 points raster scan with 1 µm increments of a *Phajus grandifolius* granule after irradiation in the centre of the mesh by a 0.3 µm beam for 30 s. The ‘pixels’ of the composite image are limited radially to the 100-reflection. The spatial extent of structural loss is schematically indicated by a circle. (*b*) The same composite image but showing integrated 100-intensity ‘pixels’. (*c*) Experimental values (rectangles and circles) and Gaussian fits (curves) of the variation of intensity across the irradiated centre. Blue curve/points: 2.5 s irradiation; red curve/points: 5 s irradiation; black curve/points: 30 s irradiation. (*d*) Width of irradiated zone (FWHM) as a function of *t* ^1/2^. A linear regression curve has been fitted to the data. Filled circles: horizontal fitted data; plus signs: vertical fitted data; empty circles: horizontal fitted data at *t* = 5 s with 60 s waiting time before start of raster-scan provide no evidence for continuing structural loss by slow reactive species.

Fig. 5(*a*) ▶ shows the composite pattern of 13 × 13 raster-scan points with 1 µm step-increments for a *Phajus grandifolius* granule which had been irradiated for 30 s by a 0.3 µm beam of about 4 × 10^10^ photons s^−1^ flux. The analogue composite pattern with the integrated 100-reflection intensity is shown in Fig. 5(*b*) ▶. The data collection time using the Medipix detector (Graceffa *et al.*, 2009 ▶) was 0.5 s per point, *i.e.* 6.5 s overall for each consecutive line-scan. The composite pattern shows that the structural loss extends rather symmetrically around the irradiated point with a width of about 7 µm. We have fitted Gaussian functions to the normalized intensity variation through the irradiated centre along a horizontal and vertical line (Fig. 5*c* ▶). The onset of radiation damage can be fitted by a single Gaussian function. The two overlapping Gaussian functions assumed for later stages provide only a semi-quantitative fit. The change in width as a function of time (≅ variable dose) has a non-linear dose dependence, which is also observed for protein crystals at RT (Blake & Phillips, 1962 ▶). We choose a *t* ^1/2^ (dose) dependence of azimuthal width but the limited amount of data points would also allow an exponential dependence (Fig. 5*d*). A similar structural loss was observed for potato starch granules which had been immersed in water (Fig. 1*a* ▶) and irradiated at selected positions by a 1 µm beam for a variable time (results not shown). In this case a linear scan was carried out across the irradiated zone using 1 µm steps between patterns and 0.1 s exposures.

We assume that the build-up of structural loss around the ∼1 µm-diameter beam track is linked to photoelectrons escaping from the beam track (Moukhametzianov *et al.*, 2008 ▶). A contribution from the tails of the focused beam on the radiation damage propagation is excluded (see also supplementary information). We also do not observe an influence of the linear polarization of the undulator on the spatial distribution of radiation damage (Nave & Hill, 2005 ▶), which suggests that the starch matrix is degraded by reactive species which spread isotropically through a diffusion process. The reactive species are probably radiolysis products such as OH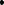 (Ward, 1988 ▶) which are known to attack polysaccharides with hydrogen-abstraction at C^1^ and C^4^ (Ershov & Isakova, 1987 ▶). The diffusion rate of the reactive species is, however, too fast to be resolved on the timescale of the line-scans. The presence of a diffusion process of radical species suggests that secondary radiation damage effects could be reduced by appropriate RT radical scavengers (Barker *et al.*, 2009 ▶).

### 3.5. Nanometre step-scanning diffraction at RT

**Figure 6:**
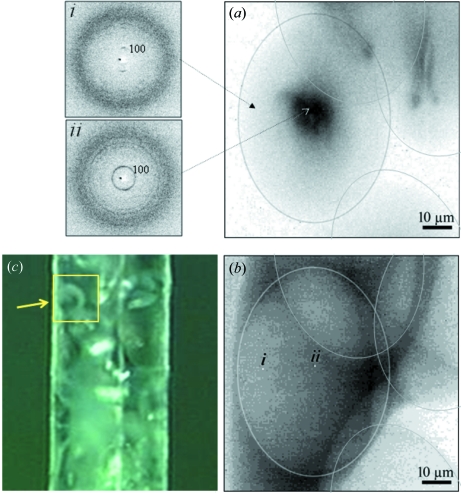
(*a*) 161 × 161 points raster microdiffraction scan of *Canna edulis* granules inside a water-filled capillary with 0.5 µm steps through a 0.3 µm × 0.3 µm beam. Patterns were collected in 0.1 s with a 2 × 2 binned FReLoN CCD. The individual ‘pixels’ of the composite pattern are scaled to the integrated relative intensity of the 100-reflection. (*b*) The same for a diffuse scattering background determined close to the 100-reflection. The outer shapes of the granules visible in (*a*) and (*b*) are indicated by elliptic boundaries. (*c*) Granules inside the capillary imaged by the beamline microscope. The region scanned is indicated with a prominent granule marked by the arrow. Two raw patterns (i, ii) are shown together with the location and dimensions of the corresponding ‘pixels’ in the composite background pattern (*b*).

The results from the previous section show that a distance of 4–5 µm is often maintained between the neighbouring points of extended raster-scans on starch granules with an exposure time of several seconds per point (Lemke *et al.*, 2004 ▶; Gebhardt *et al.*, 2007 ▶). One can, however, reduce the distance between neighbouring raster points by accepting an exposure time corresponding to a fraction of the amorphization dose. Under these conditions, however, only the strongest reflections can be analyzed for a single pattern. This can be shown for a raster scan of several *Canna edulis* granules in a water-filled glass capillary through a 0.3 µm beam. We raster-scanned 161 × 161 points with 0.5 µm step increments and 0.1 s exposure per pattern using a FReLoN CCD. Fig. 6(*a* ▶) shows a composite diffraction image composed of 161 × 161 pixels scaled to the azimuthally integrated 100-reflection intensity which allows the outskirts of four granules to be recognized (details on the integration procedure are provided in the supplementary information). The optical microscopy image is lacking in depth-of-field to clearly resolve these granules (Fig. 6*c* ▶). We have highlighted the diffraction patterns corresponding to two pixels from the centre and the rim of the prominent granule in order to show that the azimuthal width of the 100-reflection reflects the granule shell structure (Lemke *et al.*, 2004 ▶). We note that the 100-reflection intensity is not homogeneous across the prominent granule but shows a strong enhancement in its centre (Fig. 6*a* ▶). The volume-dependent diffuse scattering of the granules, determined by azimuthal integration close to the 100-reflection (Lemke *et al.*, 2004 ▶), does not, however, show the same enhancement (Fig. 6*b* ▶). A homogeneous granule density corresponds also to morphological observations (Hall & Sayre, 1970 ▶). We tentatively associate this enhancement with an increased crystallinity at the growth centre as proposed also for potato starch granules (Lemke *et al.*, 2004 ▶).

## 4. Conclusions

The current results provide evidence for primary and secondary radiation damage affecting hydrated starch granules irradiated by synchrotron radiation microbeams. Irradiation at 100 K limits primary radiation damage to the X-ray beam track as also proposed for protein microcrystallography. The propagation of radiation damage in starch granules at RT was found to be dose dependent. The formation of reactive radical species generated by radiolytic processes of photoelectrons is assumed to be at the origin of secondary radiation damage processes.

There are several possible strategies for raster-scan data collection on starch granules at RT. A frequently used strategy is to maintain the dose at each raster point close to amorphization, but to keep the distance between raster points larger than the photoelectron travel range, in order to avoid radiation damage spill-over. Alternatively, as shown in this study, one can collect data at a fraction of the local amorphization dose so that radiation damage spill-over does not significantly reduce reflection intensities at neighbouring raster points. This strategy is valuable for recording the strongest reflections with a nanometre step-resolution. Indeed, a 0.1 s pattern for a flux of 4 × 10^10^ photons s^−1^ corresponds to ∼6% of the amorphization dose of ∼1.3 photons nm^−3^ of a granule in water. Averaging of the radiation dose across neighbouring raster points also allows patterns with optimized counting statistics to be obtained. Finally, one could operate at the highest possible brilliance, possibly by an increase of band pass, and use the most sensitive detector technology with the fastest detector readout system and the highest raster speed which would allow profiting from the positive dose rate dependence of radiation damage observed for protein crystals (Southworth-Davies *et al.*, 2007 ▶). The combination of high-speed raster microdiffraction with continuous sample rotation could find use for RT protein crystallography. This option is of particular interest for raster microdiffraction experiments at the upcoming generation of ultralow-emittance third-generation synchrotron radiation sources (*e.g.* PETRA III, NSLS II, MAX IV) or the proposed energy-recovery linac sources (Bilderback *et al.*, 2010 ▶). The extent to which one can escape secondary radiation effects by staying ahead of propagating reactive species will, however, depend on the reaction rates involved which are currently not well enough known. Evidently, techniques of limiting radical propagation by radical scavengers (Barker *et al.*, 2009 ▶) should be used to enhance the potential of raster microdiffraction techniques whenever available.

## Supplementary Material

Supplementary material file. DOI: 10.1107/S0909049510028335/ms5024sup1.pdf

## Footnotes

1 Supplementary data for this paper are available from the IUCr electronic archives (Reference: MS5024). Services for accessing these data are described at the back of the journal.
