# Supplementary material for: Raster microdiffraction with synchrotron radiation of hydrated biopolymers with nanometre step-resolution: case study of starch granules
Source: J Synchrotron Radiat. 2010 Sep 3;17(Pt 6):743–50. doi: 10.1107/S0909049510028335 (PMC3025595; doi:10.1107/S0909049510028335)
Supplement: Supplementary file 1 [file s-17-00743-sup1.pdf]

Supplementary Information

Raster-microdiffraction with synchrotron radiation of hydrated biopolymers with nanometer step-resolution: case study of starch granules.

C. Riekel[1,*], M. Burghammer[1], R. J. Davies[1], E. Di Cola[1], C. König[1], H.T. Lemke[1,2], J.-L. Putaux[3] and S. Schöder[1]

[1] European Synchrotron Radiation Facility, B.P.220, F-38043 Grenoble Cedex, France

[2] Center for Molecular Movies, Niels Bohr Institute, University of Copenhagen, Universitetsparken 5, DK-2100 Copenhagen, Denmark

[3] Center de Recherches sur les Macromolécules Végétales (CERMAV-CNRS), BP 53, F-38041 Grenoble Cedex 9, France

Synchrotron radiation experiments

Synchrotron radiation (SR) experiments were performed with a monochromatic SR-beam, with a typical wavelength of ~0.1 nm from a liquid N_2 cooled Si-111 channel-cut monochromator (Riekel, 2000). The beam was focused by Kirkpatrick-Baez mirrors (Kirkpatrick & Baez, 1948) to about 1*1 μm^2 and 0.3*0.3 μm^2. The beam size was determined by knife-edge scans at the focal position. A beam divergence of ~1 mrad was obtained for a 1 μm beam through the angular acceptance of the mirrors and the collimation by slits. For experiments performed on starch granule sections, a beam size of $0.8_{hor}*0.5_{vert}$ μm^2 (full-width-at-half maximum; fwhm) was determined for a beam divergence of $1_{hor}*0.35_{vert}$ $mrad^2$ and a flux of = $3*10^{10}$ photons/sec. The flux was determined with a calibrated photodiode and was scaled to 200 mA SR ring current. For whole granule experiment a focus of $0.9_{hor}*1.2_{vert}$ μm^2 (fwhm) and a flux of = $2*10^{10}$ photons/sec were

determined (Figure S1). The Gaussian profile is, however, an approximation as shown in Figure S1 for a horizontal knife-edge scan and its derivative. Due to the presence of tails the profile of the derivative can better described by a Lorentzian function (Figure S1).

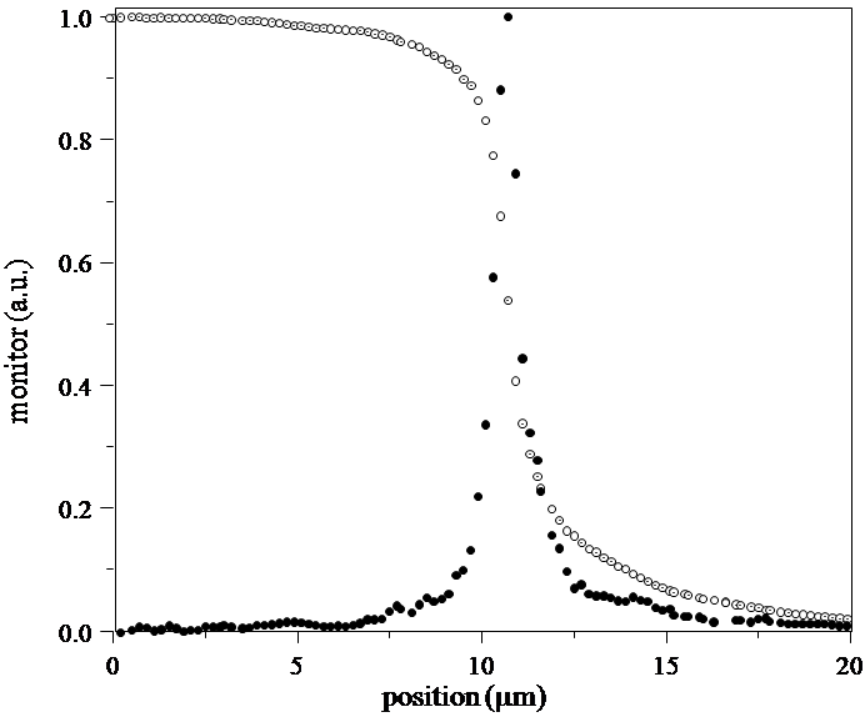

Figure S1 Horizontal knife-edge scan (open circles) and derivative (solid circles).

Selected experiments were performed with a 5 μm beam obtained by the combination of parabolic Be-refractive lenses and a Pt-aperture as collimator (Chanzy *et al.*, 2006). The beam size is in this case defined by the aperture size. A typical flux for a 5 μm beam is about 10^{11} photons/sec. The relative beam intensity was monitored during the experiments by a micro-ionization chamber in front of the sample (Kocsis & Somogyi, 2003).

The sample holder with magnetic base was attached to a Hampton Research magnetic base. For experiments with a 1 μm beam the magnetic base and sample support were attached to a microgoniometer set-up consisting of orthogonal motorized arcs or a *Kleindiek* MM3A micromanipulator (Volkringer *et al.*, 2007) which were placed on large-stroke motorized x/y/z stages. For experiments with a nanometer beam the magnetic base with the

sample was placed on a PI nanocube® (model P-615.36D) with 350x350x250 μm scan-range which is part of the ID13 nanogoniometer setup. Alignment of the sample to the beam position was carried out with a precision of about 1 μm using a calibrated Olympus microscope. The bidirectional repeatability of the sample in the 1 μm beam during mesh-scans was 100 nm vertically (MICOS GmbH UPL-160) and 200 nm horizontally (MICOS GmbH HPS-170 (MICOS specifications). Mesh-scans were performed as a series of consecutive line-scans. The marked beam position was stable for the duration of the experiment. For the PI nanocube® operating in closed-loop the repeatability was <10 nm vertically and horizontally (PI specifications).

An Oxford cryoflow system was used for flash freezing and controlling the sample temperature. For cryoflow experiments Hampton Research nylon loops were used.

Diffraction patterns were recorded using a slow readout MAR165 detector (MarResearch GmbH) or a faster readout FReLoN CCD camera (Labiche *et al.*, 2007). The 16 bit readout MAR165 detector (2048*2048 pixels of 78 μm*78 μm) was binned to 1024*1024 pixels in order to reduce the readout time. For a typical data collection time of 0.1 s the average readout time was 3.22 s which implies an average pattern repetition frequency of about 0.3 Hz. The 16 bit readout Frelon camera (2048*2048 pixels of 51 μm*51 μm) was binned to 512*512 pixels in order to increase the readout speed (about 0.7 s/pattern). For fast mapping experiments we used a Medipix2 detector with a single elements of 256*256 pixels of 55.05 μm*55.05 μm each (Ponchut *et al.*, 2002; Graceffa *et al.*, 2009). The readout speed was about 0.1 s/pattern. The exposure time was controlled for all detectors by a fast shutter. The distance of sample-to-detector was determined by Al_2O_3 or Ag-behenate calibration standards (Blanton *et al.*, 1995).

Data analysis

Diffraction patterns were displayed and analyzed using FIT2D (Hammersley, 2009) or a batch processing software for large pattern sequences (Davies, 2006). Figure S2A shows a 100 ms pattern from a *Canna edulis* granule recorded close to the growth centre. The granule was located inside a quartz capillary filled with water. No background was subtracted. The pattern corresponds to B-type starch (Buléon *et al.*, 1998). The radial profile of the 100-reflection was determined by azimuthal integration of the annulus defined by the two red rings.

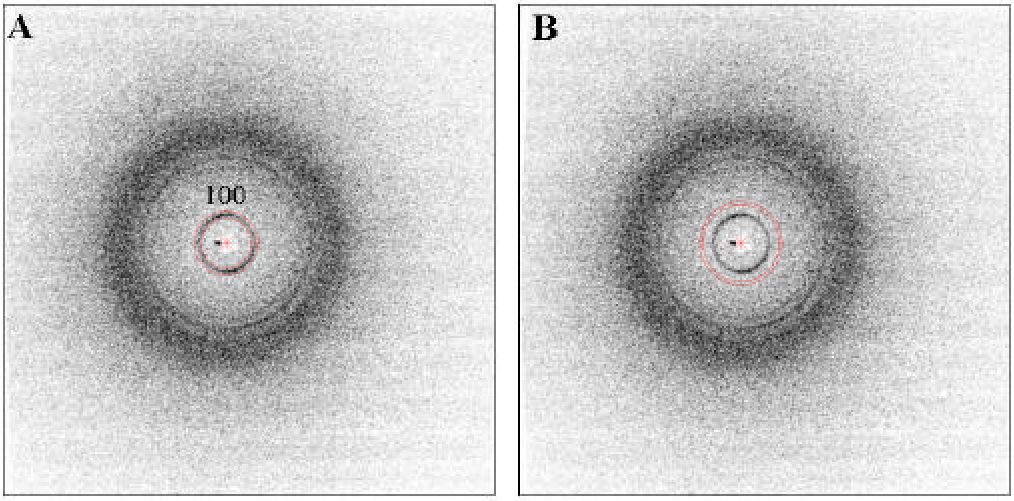

Figure S2 A: raw B-type starch diffraction pattern of Canna edulis granule in a water filled quartz capillary recorded in 100 ms by a 2x2 binned FReLoN camera. The annulus around the 100-reflection, defined by the two red rings, was azimuthally integrated. The 100-reflection intensity was determined by fitting a function the 1D-profile; B: the annulus defined by the two red rings was azimuthally integrated and radially summed in order to determine the integral background scattering.

The radial profile of the 100-reflection of selected patterns was fitted by a Voigtian function which provided a slightly better fit than a Gaussian function (χ^2-test). For recursive fits of a larger number of patterns a Gaussian profile was used. A 0-order polynomial was used as background. The annulus defined next to the 100-reflection in Figure S2B was azimuthally integrated and radially summed in order to determine the background scattering which is volume dependent (Lemke *et al.*, 2004).

The pattern of a *Phajus grandifolius* granule recorded by the Medipix2 detector is shown in Figure S3A. For better visibility of weaker reflections, 10 patterns of 0.5 s each were averaged and an averaged amorphous pattern was subtracted. No flat field correction was applied. The azimuthally averaged pattern is shown in Figure S3B.

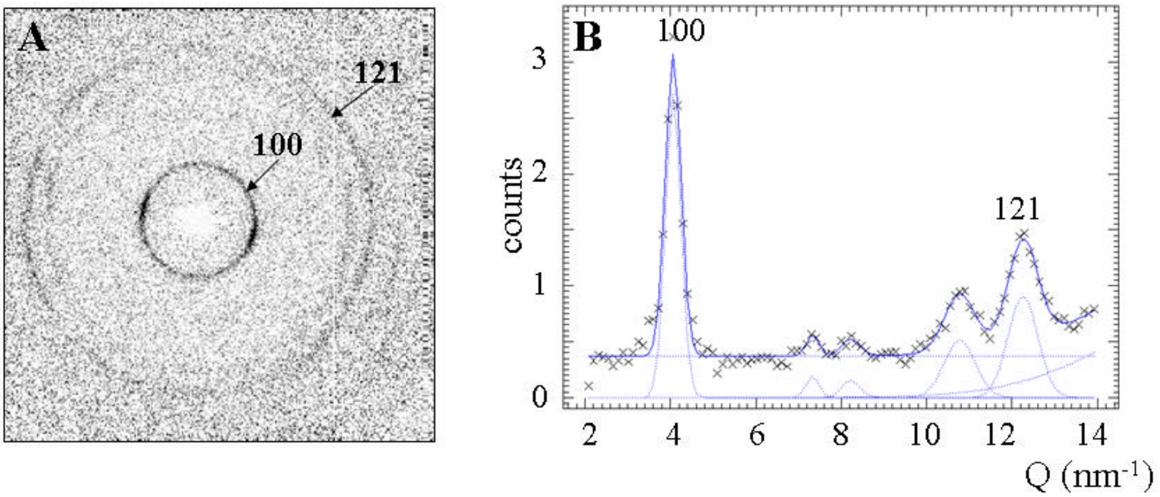

Figure S3   A: B-type starch diffraction pattern of Phajus grandifolius granule recorded by the Medipix2 detector. Ten patterns of 0.5 s each were averaged and the background subtracted; B: azimuthally integrated pattern fitted by 5 narrow Gaussian functions for the Bragg reflections, a broad Gaussian function for short-range order and a 0-order polynomial. The individual fitted peaks and the fitted profile are shown in blue.

The pattern was fitted by 5 Gaussian functions for the Bragg peaks, a broad Gaussian function for the short range order background and a 0-order polynomial for the residual background scattering.

The variation of the intensity across horizontal and vertical lines through the irradiated centre of a *Phajus grandifolius* granule (Figure 3A,B of article) was fitted with Gaussian functions in order to determine the spatial extent of structure loss. We subtracted the maximum intensity in each set of data points and multiplied the data points by -1. The thus normalized data were fitted by either one Gaussian function (Figure S4: blue/red curves) or two Gaussian function (Figure S4: black curve).

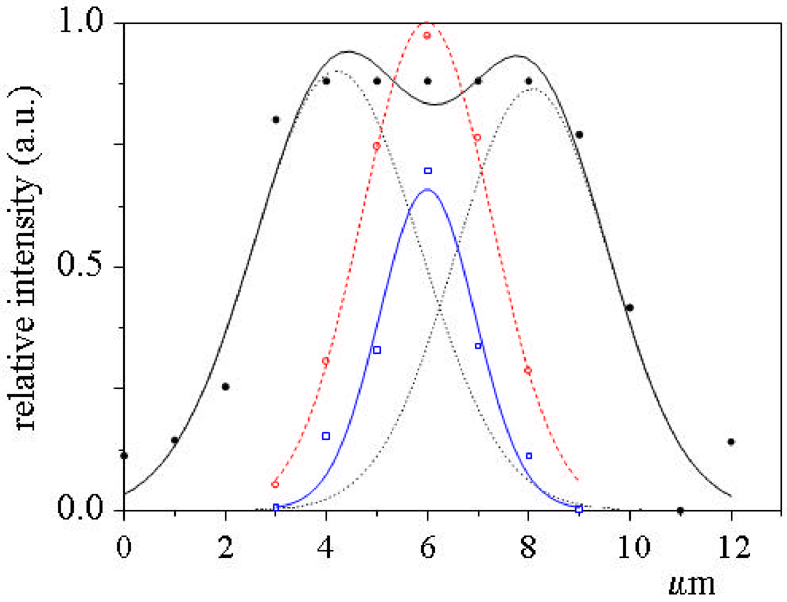

Figure S4 Fit of Gaussian functions to the variation of intensity on a horizontal line through the irradiated centre of a Phajus grandifolius granule; experimental values (rectangles and circles) and Gaussian fits (curves) of intensity profile; blue curve/points: 2.5 s irradiation; red curve/points: 5 s irradiation; black curve/points: 30 s irradiation;

The variation of the azimuthal width of the 100-reflection for a horizontal sequence of patterns through the growth centre of the granule (dashed line in Figure 4B of article) was determined by fitting a Lorentzian function to each reflection profile, which was radially averaged across the reflection. The dashed curve shown in Figure S5 of this note is based on a Lorentzian fit of experimental values for a whole granule (Lemke *et al.*, 2004).

Radiation dose calculation

For an averaged 1.1 µm diameter SR-beam impinging at the centre a 50 µm diameter granule the irradiated volume is $3.9*10^{10}$ nm^3. For a flux of $1.8*10^{10}$ photons/s and complete amorphisation after 3.7 s irradiation one calculates a dose of 1.3 photons/nm^3.

Laser micro-dissection of starch granules

Air-dry potato starch granules of 70 µm (average size; obtained by sieving) were attached to a glass cover slide using a thin film of bees' wax. The glass cover slide was placed on top of

a glass slide with the wax layer face down, separated using double sided adhesive tape. This produced a gap between the two slides, big enough for the starch granules. The granules were prepared using a pulsed UV laser micro-dissection system (PALM Microlaser Technologies GmbH, Bernried, Germany) installed at a Zeiss Axiovert 200M inverted microscope. This is particularly applicable to biomaterials such as chitin (Seidel *et al.*, 2008) as cutting is achieved by ablation. Local structural damage is therefore limited and there are no problems associated with sample heating. This is corroborated by scanning the edge of a Kevlar[149] fiber through an about 1 μm synchrotron radiation (SR) beam. The WAXS experiment did not show any degradation at the edge of the micro-dissected fiber such as additional diffuse scattering or azimuthal reflection broadening (unpublished data).

The geometry chosen provides enough space for the debris generated during sectioning to be distributed without blocking the laser beam and to maintain an upright position of the granule. The size of the granules did not allow them to be cut in a single step. It was therefore necessary to perform repetitive sectioning in steps of increasing focal position, often 30-60 times. This allowed the sectioning of a whole granule down to about 12 μm, which corresponds to an aspect ratio of about 6:1 for a 70 μm sized granule. In principle, the ≤1 μm laser beam focus allows higher aspect ratios to be reached, but the current limit is imposed by handling of the fragile samples (see below). The use of optical tweezers for sample manipulation (Cojoc *et al.*, 2007) in combination with laser micro-dissection is a promising possibility for obtaining granule sections with larger aspect ratios.

After cutting, the granule section was hydrated by transferring it into a drop of deionised water on a glass slide for about 30 minutes. It was then pushed gently by a 10 μm thick nylon fiber into a neighbouring drop of 30% glycol/water solution and picked-up by a Hampton Research nylon loop. The glycol/water solution allows flash-freezing of the sample which limits secondary radiation damage in SR-experiments, as for SR-protein crystallography

(Lemke *et al.*, 2004). The transfer of the granule section between the liquids and into the nylon loop was found to be the most delicate step and easily resulted in breaking part of the section away. Subsequent experiments were therefore carried out using a 25 μm thick granule section with an aspect ratio of about 3:1, which proved to be sufficiently stable during all operations.

In order to test the influence of the residual shell structure on the patterns (Lemke *et al.*, 2004) we have determined the azimuthal width of the 100 reflection for a line through the growth centre (Figure S5). Most values are in the range 20°< fwhm <40° (fwhm: full-width-half-maximum) set aside two "spikes" with values = 60° fwhm, which are close to the disordered growth centre. The increase of the width of the fitted Lorentzian curve from the edge (≈ 20°) to the centre (≈ 40°) is due to the finite thickness of the granule section, which results in a width-contribution of from curved layers to the chain orientation distribution within the gauge volume (Lemke *et al.*, 2004).

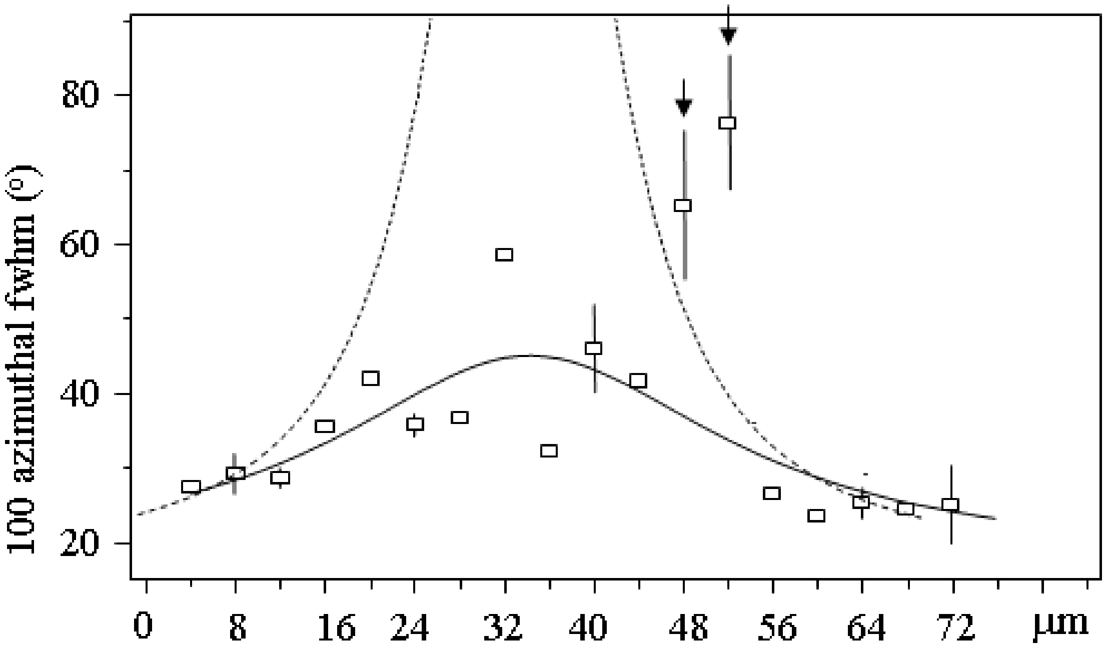

Figure S5 Azimuthal width of 100-reflections derived from patterns recorded on a line through the growth centre of the granule section (see Figure 4B of main text.) A Lorentzian curve has been fitted through the data point excluding the ones indicated by vertical arrows. Error bars (standard deviation) of selected data points are indicated. For comparison a Lorentzian fit curve based on experimental data from a whole granule is shown (barred curve).

For an idealized granule model of concentric shells with double-helical chains oriented normal to the shell surfaces and a homogeneous crystallinity, the maximum angular spread (α) of double-helices relative to the radial direction is reached at the granule centre with $\alpha = \pm 90°$ (Lemke *et al.*, 2004). The dashed curve shown in Figure S5 is based on this model and has been constrained to 180° fwhm at the centre ($\alpha = \pm 90°$) (Lemke *et al.*, 2004). The width contribution from a 25 µm thick section in the centre is according to this model: $\alpha \leq \pm 10°$. Set aside the "spikes", the azimuthal width values for the section are therefore systematically lower than for a whole granule. Further improvement of the laser microdissection technique should allow reducing the thickness of the granule section for a SR-experiment. By scanning a perfectly aligned section(Davies *et al.*, 2008) through an about 100 nm focal spot (Schroer *et al.*, 2008), the model of alternating 120-400 nm thick amorphous and semicrystalline layers (Buléon *et al.*, 1998) could be tested by SAXS/WAXS and complementary techniques.

References

Blanton, T. N., Huang, T. C., Toraya, H., Hubbard, C. R., Robie, S. B., Louer, D., Goebel, H. E., Will, G., Gilles, R. & Raftery, T. (1995). Powder Diffraction **10**, 91-95.

Buléon, A., Colonna, P., Planchot, V. & Ball, S. (1998). Int. J. Biol. Macrom. **23**, 85-112.

Chanzy, H., Putaux, J. L., Dupeyre, D., Davies, R., Burghammer, M., Montanari, S. & Riekel, C. (2006). J. Struct. Biol. **154**, 100-110.

Cojoc, D., Ferrari, E., Garbin, V., Fabrizio, E. D., Amenitsch, H., Rappolt, M., Sartori, B., Laggner, P., Burghammer, M. & Riekel, C. (2007). Appl. Phys. Lett. **91**, 234107.

Davies, R. (2006). J. Appl. Cryst. **39**, 267-272.

Davies, R. J., Koenig, C., Burghammer, M. & Riekel, C. (2008). Appl. Phys. Lett. **92**, 101903-101901.

Graceffa, R., Burghammer, M., Davies, R., Ponchut, C. & Riekel, C. (2009). Appl. Phys. Lett. **94**, 062902.

Hammersley, A. (2009). The FIT2D Home Page, www.esrf.fr/computing/scientific/FIT2D/, Grenoble: ESRF.

Kirkpatrick, P. & Baez, A. V. (1948). J. Opt. Soc. Am. **38**, 766.

Kocsis, M. & Somogyi, A. (2003). J. Synchotron Rad. **10**, 187-190.

Labiche, J. C., Mathon, O., Pascarelli, S., Newton, M. A., Ferre, G. G., Curfs, C., Vaughan, G. & Homs, A. (2007). Rev. Sci. Instrum. **78**, 091301.

Lemke, H., Burghammer, M., Flot, D., Roessle, M. & Riekel, C. (2004). Biomacromolecules **5**, 1316-1324.

Ponchut, C., Visschers, J. L., Fornaini, A., Graafsma, H., Maiorino, M., Mettivier, G. & Calvet, D. (2002). Nucl. Instrum. Methods A **484**, 396-406.
Riekel, C. (2000). Rep. Prog. Phys. **63**, 233-262.
Schroer, C. G., Boye, P., Feldkamp, J., Patommel, J., Schropp, A., Schwab, A., Stephan, S., Burghammer, M., Schoeder, S. & Riekel, C. (2008). Phys. Rev. Lett. **101**, 090801.
Seidel, R., Gourrier, A., Burghammer, M., Riekel, C., Jeronimidis, G. & Paris, O. (2008). Micron **39**, 198-205.
Volkringer, C., Popov, D., Loiseau, T., Guillou, N., Ferey, G., Haouas, M., Taulelle, F., Mellot-Draznieks, C., Burghammer, M. & Riekel, C. (2007). Nature Materials **6**, 760 - 764.
